# Efficacy of combined gemcitabine, oxaliplatin and pegaspargase (P-gemox regimen) in patients with newly diagnosed advanced-stage or relapsed/refractory extranodal NK/T-cell lymphoma

**DOI:** 10.18632/oncotarget.8647

**Published:** 2016-04-08

**Authors:** Jing-hua Wang, Liang Wang, Cheng-cheng Liu, Zhong-jun Xia, Hui-qiang Huang, Tong-yu Lin, Wen-qi Jiang, Yue Lu

**Affiliations:** ^1^ Department of Hematologic Oncology, Sun Yat-Sen University Cancer Center, Guangzhou, 510060, People's Republic of China; ^2^ State Key Laboratory of Oncology in South China, Collaborative Innovation Center for Cancer Medicine, Guangzhou, 510060, People's Republic of China; ^3^ Collaborative Innovation Center for Cancer Medicine, Guangzhou, 510060, People's Republic of China; ^4^ Department of Medical Oncology, Sun Yat-Sen University Cancer Center, Guangzhou, 510060, People's Republic of China

**Keywords:** extranodal NK/T-cell lymphoma, gemcitabine, oxaliplatin, pegaspargase, adverse effects

## Abstract

Extranodal natural killer/T-cell lymphoma (ENKTL) is an aggressive neoplasm with a poor outcome. Asparaginase-based regimens are recommended for patients with advanced-stage or relapsed/refractory ENKTL. We retrospectively investigated the efficacy and toxicity of combined gemcitabine, oxaliplatin, and pegaspargase (P-gemox) in these patients. A total of 35 patients with newly diagnosed stage III–IV, relapsed or refractory ENKTL were treated with 2 to 8 cycles of P-gemox: gemcitabine (1250 mg/m^2^) and oxaliplatin (85 mg/m^2^) injected intravenously and pegaspargase (2500 IU/m^2^) injected intramuscularly on day 1 and repeated every 2 weeks. Upon completion of treatment, the overall response rate was 80.0%, with a complete response in 51.4% of patients. The 1-, 2- and 3- year progression-free survival rates were 45.0%, 38.6% and 38.6%, and overall survival rates were 76.8%, 64.7% and 64.7%, respectively. Patients who attained a complete response showed better progression-free survival than those without a complete response (*p* = 0.01). The major adverse effects were hematologic toxicity and liver dysfunction. Grade 3/4 leucopenia and neutropenia occurred in 40.0% of patients. No treatment-related deaths occurred. These results indicate the P-gemox regimen is a safe and effective treatment for patients with newly diagnosed advanced-stage or relapsed/refractory ENKTL. We anticipate future prospective trials will confirm the efficacy.

## INTRODUCTION

Extranodal natural killer (NK)/T-cell lymphoma (ENKTL) is a distinct clinicopathological entity in the WHO classification of lymphoid neoplasms [1]. ENKTL is diagnosed more commonly in Asia and Latin America than in Western countries [2] and accounts for 5%–10% of all malignant lymphomas in China [3]. In more than 80% of cases, ENKTL originates in the nasal and upper airway region [2, 4, 5]. However, a similar disease also arises at extranasal sites, such as the skin, soft tissue, gastrointestinal tract, testis, and brain [2, 5, 6]. ENKTL is a highly invasive tumor with a short doubling time and poor prognosis. Because of its rarity, limited geographic distribution, and paucity of multicenter prospective clinical trials, the optimal first-line therapy for ENKTL continues to evolve. Systemic chemotherapy combined with involved-field radiotherapy is recommended for localized ENKTL [7] and yields a long-term survival rate of 70–80% [8, 9]. Patients with advanced-stage or relapsed/refractory ENKTL follow an extremely aggressive clinical course [10], and chemotherapy remains the mainstay treatment. However, the conventional chemotherapy regimens based on CHOP (cyclophosphamide, doxorubicin, vincristine, and prednisone) give unsatisfactory responses, and survival beyond 1 year is extremely rare among patients with advanced-stage ENKTL [2, 11–13]. The poor outcome is in part because ENKTL tumor cells express P-glycoprotein, which mediates tumor multidrug resistance (MDR) [14–16].

L-Asparaginase has a unique anti-tumor mechanism that is not affected by P-glycoprotein: tumor cells unable to synthesize L-asparagine die when their stores of L-asparagine are depleted by L-asparaginase [17, 18]. Moreover, L-asparaginase exhibits anti-tumor activity against NK cell tumors *in vitro* and *in vivo* [17, 18]. The first study of the clinical efficacy of L-asparaginase in refractory or relapsed ENKTL was published by Yong et al. in 2000 [19]. Since then, additional L-asparaginase-based regimens, like combined dexamethasone, methotrexate, ifosfamide, L-asparaginase, and etoposide (SMILE) [20, 21] and combined asparaginase, methotrexate, and dexamethasone (AspaMetDex) [22], have emerged as promising treatments for advanced-stage and relapsed/refractory ENKTL. However, the SMILE regimen is associated with unacceptable toxicities, and the efficacy of the AspaMetDex regimen requires further investigation in additional clinical trials. At our center, Wang et al. attempted to use a combined gemcitabine, oxaliplatin and L-Asparaginase (GELOX) regimen for stage IE/IIE ENKTL [23]. None of the agents included in the GELOX regimen were affected by P-glycoprotein, and it yielded promising results. Guo et al. also demonstrated the efficacy of this regimen [24]. However, the efficacy of this regimen against advanced-stage and relapsed/refractory ENKTL is not clear.

Pegylated asparaginase (pegaspargase), a form of *Escherichia coli* L-asparaginase that is covalently linked to polyethylene glycol, has proven effective against acute lymphoblastic leukemia and ENKTL with less toxicity and a longer half-life than free L-asparaginase [25, 26]. We therefore conducted a retrospective study to evaluate the treatment outcomes and safety profile of a P-gemox regimen composed of gemcitabine, oxaliplatin and pegasparase for newly diagnosed advanced-stage and relapsed/refractory ENKTL cases at a single institution.

## RESULTS

### Patient characteristics

The patient characteristics are listed in Table 1. The median age was 38 years (range, 16 to 65 years), and the male:female ratio was about 2:1. Nineteen patients were newly diagnosed with advanced-stage ENKTL, and 16 had relapsed/refractory disease. The primary involvement site was the upper aerodigestive tract (nasal cavity, nasopharynx and tonsils) in 24 patients. Twenty-one patients (60%) had systemic B symptoms, and 15 patients (42.8%) had elevated levels of LDH. The distribution of IPI scores included 10 patients (28.6%) with IPI scores of 0–1 (low-risk group) and 25 (71.3%) with IPI scores of 2–4 (intermediate-risk or high-risk group). For relapsed/refractory patients, previous treatments included anthracycline-containing regimens (CHOP or CHOP-like regimens) alone (*n* = 9) or followed by radiotherapy (*n* = 4), a concomitant 2/3DeVIC regimen (dexamethasone, etoposide, ifosfamide, and carboplatin) or a VIPD regimen (etoposide, ifosfamide, cisplatin, and dexamethasone) and radiotherapy (*n* = 2), and radiotherapy alone (*n* = 1).

**Table 1:** Characteristics of 35 patients with ENKTL treated with P-gemox

Characteristic	No. of Patients	%
Age, years		
18–30	13	37.1
31–50	15	42.9
> 50	7	20.0
Gender		
Male	23	65.7
Female	12	34.3
B symptoms		
Present	21	60
Absent	14	40
Primary site		
Upper aerodigestive tract	24	68.6
Extra-upper aerodigestive tract	11	31.4
ECOG PS		
0	25	71.4
1	10	28.6
Disease status		
Newly diagnosed	19	54.3
Relapsed/Refractory	16	45.7
Ann Arbor stage		
I/II	8	22.8
III/IV	27	77.1
IPI score		
0	5	14.3
1	5	14.3
2	13	37.1
3	11	31.4
4	1	2.8
KPI score		
0	3	8.6
1	5	14.3
2	18	51.4
3	6	17.1
4	6	8.6
sLDH		
Normal	17	48.6
Increased	15	42.8

Abbreviations and note: P-gemox, gemcitabine, oxaliplatin and pegaspargase; ECOG PS, Eastern Cooperative Oncology Group performance status; IPI, International Prognostic Index; sLDH, serum lactate dehydrogenase; B symptoms include unexplained fever with temperature above 38°C, night sweats or weight loss more than 10% within 6 months.

### Treatment and response

The total cycles of P-gemox regimen received by all patients were 157, with a median of 5 cycles per patient (range, 2–8 cycles), and pegaspargase was administrated in 155 of the 157 cycles. During the treatment, four patients went off the treatment because of disease progression or severe toxicity, and six patients had a dose reduction or delay because of severe hematological toxicity (*n* = 3), abnormal liver function (*n* = 1) or hypofibrinogenemia (*n* = 2).

At the interim assessment after 2 cycles of P-gemox, there were 9 CRs (25.7%), 24 PRs (68.6%), 1 SD (2.9%) and 1 PD (2.9%). Eleven patients received additional primary involved-field radiotherapy at a median dose of 50 Gy after chemotherapy. Seven patients underwent ASCT after achieving interim CR or PR. As shown in Table 2, upon treatment completion, the CR rate was improved to 51.4% (18 of 35 patients), and the PR rate was 28.6% (10 of 35 patients), yielding an ORR of 80.0%. On the other hand, one patient showed SD (2.9%), and six patients had PD (17.1%). Of the 19 patients with newly diagnosed advanced-stage ENKTL, the ORR after P-gemox chemotherapy was 78.9%, with a CR rate of 42.1%. Similarly, of the 16 patients with relapsed or refractory ENKTL, the ORR was 81.3% with a CR rate of 62.5%. There were no differences in either the ORR or CR rate between patients with newly diagnosed advanced-stage disease and those with relapsed/refractory disease. There were no significant clinicopathologic factors associated with CR or ORR (data not shown).

**Table 2:** Rates of responses to the P-gemox regimen by the disease state

Response	All Patients (*N* = 35)	Newly diagnosed advanced-stage (*n* = 19)	Relapsed/Refractory (*n* = 16)
CR (%)	18 (51.4)	8 (42.1)	10 (62.5)
PR (%)	10 (28.6)	7 (36.8)	3 (18.8)
SD (%)	1 (2.9)	1 (5.3)	0 (0)
PD (%)	6 (17.1)	3 (15.8)	3 (18.8)
ORR (%)	28 (80.0)	15 (78.9)	13 (81.3)

Abbreviations: P-gemox, gemcitabine, oxaliplatin and pegaspargase; CR, complete response; PR, partial response; SD, stable disease; PD, progressive disease; ORR, overall response rate.

### Survival

**Figure 1:**
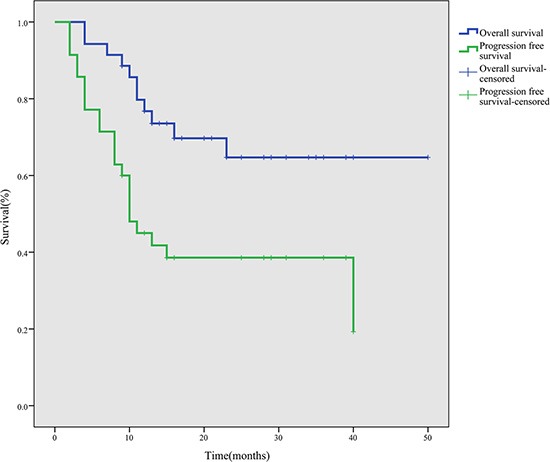
Survival curves for 35 ENKTL patients treated with the P-gemox regimen

After a median follow-up period of 28 months (range, 9–50 months), the 1-, 2- and 3-year PFS rates were 45.0%, 38.6% and 38.6%, and the OS rates were 76.8%, 64.7% and 64.7%, respectively (Figure 1). During the follow-up period, 11 patients died due to disease progression.

**Figure 2:**
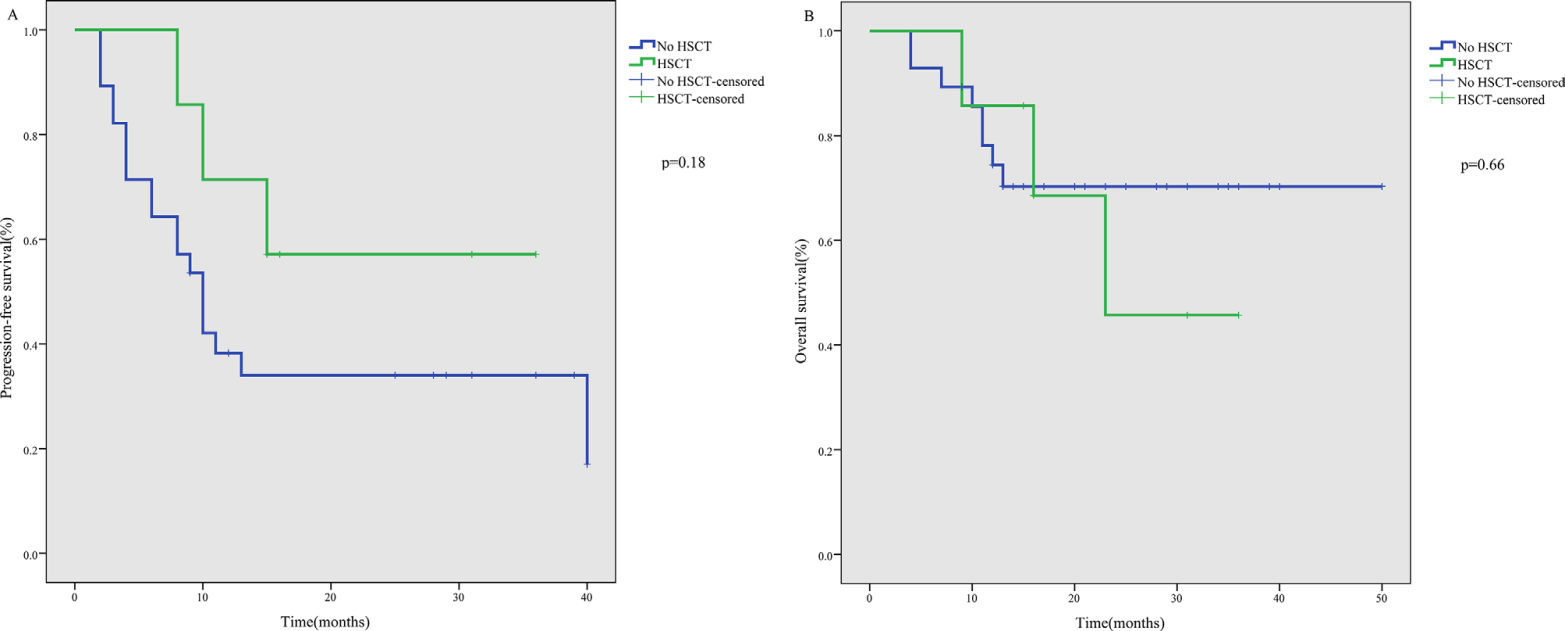
PFS (**A**) and OS (**B**) after P-gemox regimen chemotherapy according to HSCT.

Of the seven patients who received ASCT, four were still alive with a persistent CR, while three relapsed and died due to disease progression by the end of follow-up. The median PFS and OS of these seven patients were 15 and 16 months, respectively. Patients who received ASCT tended to show better PFS (*p* = 0.18) and OS (*p* = 0.66), but the difference was not statistically significant (Figure 2).

### Adverse effects

Treatment-related adverse effects are shown in Table 3. The most common adverse effect of the P-gemox regimen was hematological toxicity. However, non-hematologic toxicities were also frequent, though most reported cases were mild in severity. Mild coagulation abnormalities, which were reflected by prolongation of the activated partial thromboplastin time (APTT), were observed in 17 patients. Seven patients developed fibrinogen deficiencies, most of which were grade 1/2. Hepatic transaminase elevation was seen in 85.7% of the patients, but most were grade 1/2. After administration of symptomatic treatment, liver function and coagulation abnormalities recovered to normal or near normal before the next cycle of chemotherapy. Serious gastrointestinal toxicity was infrequent, and there were only two (5.8%) patients who presented with grade 3/4 nausea, emesis or diarrhea. All adverse events were manageable with supportive care. One patient developed left upper limb deep venous thrombosis and revascularization was achieved following treatment using an anticoagulant. Other noticeable toxicities included hypertriglyceridemia, hyperglycemia, hypoglycemia and hypoalbuminemia. No allergic reactions or pancreatitis were observed. No treatment-related deaths occurred.

**Table 3:** Adverse effects of P-gemox chemotherapy

Toxicity	Toxicity Incidence, No. (%)					
	Grade 0	Grade 1	Grade 2	Grade 3	Grade 4	Total
Hematologic toxicity						
Leukopenia	3 (8.6)	6 (17.1)	12 (34.3)	11 (31.4)	3 (8.6)	32 (91.4)
Neutropenia	6 (17.1)	4 (11.4)	11 (31.4)	11 (31.4)	3 (8.6)	29 (82.9)
Anemia	1 (2.9)	11 (31.4)	14 (40.0)	7 (20.0)	2 (5.7)	34 (97.1)
Thrombocytopenia	17 (48.6)	4 (11.4)	3 (8.6)	5 (14.3)	6 (17.1)	18 (51.4)
Gastrointestinal disorders						
Nausea, vomiting, diarrhea	22 (62.9)	5 (14.3)	6 (17.1)	1 (2.9)	1 (2.9)	13 (37.1)
Liver dysfunction						
Increased transaminases	5 (14.3)	18 (51.4)	8 (22.9)	3 (8.6)	1 (2.9)	30 (85.7)
Hyperbilirubinemia	28 (80.0)	4 (11.4)	1 (2.9)	1 (2.9)	1 (2.9)	7 (20.0)
Coagulopathy						
APTT elongation	18 (51.4)	15 (42.9)	2 (5.7)	0 (0.0)	0 (0.0)	17 (48.6)
Hypofibrinogenemia	20 (57.1)	5 (14.3)	7 (20.0)	3 (8.6)	0 (0.0)	15 (42.9)
Increase in BUN	30 (85.7)	5 (14.3)	0 (0.0)	0 (0.0)	0 (0.0)	5 (14.3)
Hypertriglyceridemia	8 (22.9)	16 (45.7)	4 (11.4)	3 (8.6)	4 (11.4)	27 (77.1)
Hyperglycemia	15 (62.5)	5 (20.8)	3 (12.5)	1 (4.2)	0 (0.0)	20 (57.1)
Hypoglycemia	15 (62.5)	9 (37.5)	0 (0.0)	0 (0.0)	0 (0.0)	20 (57.1)
Hypoalbuminemia	5 (14.3)	10 (28.6)	19 (54.3)	1 (2.9)	0 (0.0)	30 (85.7)

Abbreviations: P-gemox, gemcitabine, oxaliplatin and pegaspargase; APTT, activated partial thromboplastin time.

## DISCUSSION

For patients with advanced stage, relapsed, or refractory ENKTL, chemotherapy is a mainstay treatment. This study demonstrates that the P-gemox regimen is an effective and tolerable treatment strategy for this patient population. A number of L-asparaginase-based regimens have yielded promising results in patients with advanced stage or relapsed/refractory ENKTL in recent years [20, 22, 27–29]. Moreover, pegaspargase is associated with a lower incidence of anti-asparaginase antibody induction and exhibits more prolonged asparaginase activity than native L-asparaginase [30]. Several case reports and clinical studies suggest pegaspargase is a potentially effective agent when employed in the treatment of ENKTL [23, 29, 31, 32]. Gemcitabine, which is widely used to treat solid tumors, is effective against lymphoma, particularly in relapsed/refractory patients [33–35]. Oxaliplatin is also effective against non-Hodgkin lymphoma, and the objective response rate of its single-drug activity can reach 27% to 40% [36]. When gemcitabine and oxaliplatin are used in combination, a promising synergistic effect has been observed *in vitro* and in clinical studies with lymphoma [37, 38]. In a previous phase II clinical trial to evaluate the efficacy and safety of GELOX as induction therapy for early stage ENKTL, we observed a 3-year OS rate of 78.0% with acceptable safety profiles [23]. In the present study, we retrospectively evaluated the efficacy and safety of the P-gemox regimen in the treatment of newly diagnosed advanced-stage, relapsed or refractory ENKTL. L-asparaginase in the GELOX regimen was replaced for pegaspargase in the P-gemox regimen to achieve more prolonged continuous asparagine depletion as well as easier administration, as only a single treatment every 2 weeks is required.

In recent years, additional L-asparaginase-based regimens, such as SMILE and AspaMetDex, have yielded promising results in advanced-stage and relapsed/refractory ENKTL. Table 4 summarizes the latest published results from L-asparaginase-based regimens for this disease. From this table, we can see that the treatment responses and survival outcomes obtained with the P-gemox regimen are superior or similar to those achieved with the SMILE [20, 21] and AspaMetDex [22] regimens, which is indicative of the excellent antitumor effect of the P-gemox regimen.

**Table 4:** Comparison of pegaspargase- or L-asparaginase-based regimens in the treatment of extranodal NK/T-cell lymphoma

Disease status	No.	Treatment	Response		Survival		Adverse effects		Reference
			ORR	CR	OS	PFS	Grade 3/4 neutropenia	Grade 3/4 hepatotoxicity	
Newly diagnosed, advanced-stage or relapsed/refractory	35	P-gemox ±sandwiched RT (50 Gy)	80.0%	51.4%	1 y: 76.8%	1 y: 45.0%	40%	11.5%	this study
Newly diagnosed, relapsed/refractory, any stage	87	SMILE ±sandwiched RT (50 Gy)	81%	66%	5 y: 50%	4 y DFS: 64%	67%	7%	21
Newly diagnosed, stage IV, or relapsed/refractory	38	SMILE	79%	45%	1 y: 55%	1 y: 53%	100%	32%	20
Relapsed/refractory	19	AspaMetDex	78%	61%	2 y: 40%	2 y: 40%	42%	16%	22

Abbreviations: No., number; ORR, overall response rate; CR, complete remission; OS, overall survival; PFS, progression-free survival; DFS, disease-free survival; RT, radiotherapy; y, year; P-gemox, gemcitabine, oxaliplatin and pegaspargase; SMILE, methotrexate, ifosfamide, dexamethasone, etoposide, and L-asparaginase; AspaMetDex, L-asparaginase, methotrexate, and dexamethasone; GELOX, gemcitabine, oxaliplatin, and L-asparaginase.

Interestingly, the responses of patients receiving P-gemox as upfront therapy for newly diagnosed advanced-stage ENKTL were similar to those of patients receiving P-gemox as salvage for relapsed/refractory disease. This is usually not the case. Most other chemotherapeutic regimens are more effective in newly diagnosed than relapsed/refractory patients, probably due to P-glycoprotein-mediated MDR. As mentioned above, pegaspargase is not affected by MDR and exhibits a unique anti-tumor mechanism: tumor cells unable to synthesize asparagine die when their stores of asparagine are depleted by L-asparaginase. It will thus exhibit similar efficacy in all patients not previously treated with P-gemox. In addition, the patients who had a CR showed better PFS than those who did not, suggesting that for patients without a CR to our treatment, alternative treatments will be needed to prevent relapses.

Among the seven patients who received ASCT, four were alive with persistent CR, while three relapsed and died of their disease by the end of follow-up. Because of the relatively small sample size, it was not possible to draw a robust conclusion that ASCT consolidation improves survival. Although ASCT has been evaluated in many studies of ENKTL patients, and appears to benefit patients with advanced stage disease [39–41], the impact of ASCT in the era of asparaginase-based treatments remains unknown. Therefore, international prospective trials are needed to address issues around the benefits and timing of ASCT for the treatment of ENKTL.

Among the adverse events reported, grade 3/4 toxicities were mainly hematological. Timely administration of G-CSF was essential when patients experienced severe cytopenias after chemotherapy. Other commonly reported side effects of pegaspargase therapy include gastrointestinal disorders, liver dysfunction, coagulopathies, hypertriglyceridemia, hyperglycemia, and hypoalbuminemia [42]. In the present study, grade 1–2 toxicities were frequently related to these side effects, and could be well controlled with supportive treatments. Venous thrombosis is another known adverse event of asparaginase, and one patient developed left upper limb deep venous thrombosis during our study period, but revascularization was achieved following anticoagulant treatment. Thus, patients must be monitored closely for thrombosis during follow-up and in subsequent trials. There were no allergic reactions or pancreatitis observed in this study, and no treatment-related deaths occurred. The hematologic toxicities and liver dysfunction seen in our study were considerably milder than those reported with the SMILE and AspaMetDex regimens (Table 4). In sum, the P-gemox regimen presented acceptable toxicity profiles.

In conclusion, this study demonstrated that P-gemox is an effective and well-tolerated treatment for patients with newly diagnosed advanced-stage or relapsed/refractory ENKTL. However, this is a retrospective nature study with a relatively small sample size. Prospective randomized clinical trials will be needed to validate the long-term efficacy of the P-gemox regimen.

## MATERIALS AND METHODS

### Patients

From October 2010 to November 2014, a total of 35 patients with newly diagnosed stage III to IV, relapsed or refractory ENKTL who received the P-gemox regimen in Sun Yat-sen University Cancer Center were identified and included in this retrospective analysis. Clinical information was obtained through a review of medical records. The inclusion criteria for this retrospective study were as follows: (1) pathologically confirmed diagnosis of ENKTL based on the WHO “classification of Tumors of Hematopoietic and Lymphoid Tissues”; (2) Ann Arbor stage III to IV or relapsed/refractory disease; (3) no previous or concomitant malignancies; and (4) a complete set of clinical information and follow-up data. This study design was approved by the SunYat-sen University Cancer Center Research Ethics Board. Informed consent for the use and publication of medical information was obtained from all patients during their first visit.

Patients were staged on the basis of Ann Arbor staging system; International Prognostic Index (IPI) scores were calculated on the basis of age, Eastern Cooperative Oncology Group performance status (ECOG PS), Ann Arbor stage, serum biochemistry with lactate dehydrogenase (LDH), and the number of extranodal extensions. Positron emission tomography (PET)-CT scans, bone marrow Epstein-Barr virus-encoded small RNA (EBER) stain, and Epstein-Barr virus (EBV) DNA blood levels were not included in the routine staging investigations in the study. Follow-up information was obtained from patients' medical records or by telephone.

### Treatment

Patients were treated using the P-gemox regimen, which included gemcitabine (1250 mg/m^2^) and oxaliplatin (85 mg/m^2^) injected intravenously and pegaspargase (2500 IU/m^2^) injected intramuscularly on day 1 and repeated every 2 weeks. A maximum of 6 to 8 chemotherapy cycles were administered. During the chemotherapy intervals, patients with hematologic toxicities leading to a white blood cell count < 2.0 × 10^9^/L or absolute neutrophil count ≤ 1.0 × 10^9^/L received granulocyte-colony-stimulating factor (G-CSF) (5 μg/kg/day) until their neutrophil counts had recovered. For patients who had never been irradiated, IFRT for the primary anatomic site or residue lesion was administered after finishing all chemotherapy treatments. In addition, patients could receive autologous stem cell transplantation (ASCT) after achieving complete remission or partial remission. The decision was made according to the discretion of the treating physicians, mainly on the basis of the patient's age, comorbidities, and patient's wishes.

### Responses and toxicity criteria

Teatment responses were assessed after every two cycles of chemotherapy and classified as complete response (CR), partial response (PR), stable disease (SD), or progressive disease (PD) according to the Revised Response Criteria for Lymphoma [43]. The overall response rate (ORR) was defined as the proportion of patients who achieved a CR or PR. Interim response was assessed after two courses, and the completion response was defined as the response after the last course of P-gemox. Patients achieving an interim CR or PR continued to receive P-gemox until ASCT, relapse, or completion of all courses. Patients with SD on interim assessment may have continued to receive P-gemox, whereas patients with PD received other salvage regimens.

After the treatment was completed, patients were followed up and assessed by their oncologist in the outpatient department. Each assessment consisted of a physical examination, complete blood count, serum biochemistry (including LDH level), and either a CT scan, MRI of the involved region or PET-CT scan. Toxicity after chemotherapy was evaluated according to the National Cancer Institute Common Terminology Criteria of Adverse Events v3.0.

### Statistical analysis

The primary end point was ORR at the end of treatment. The secondary end points were CR, overall survival (OS), progression-free survival (PFS) and toxicity. OS was measured from the date of the first administration of the P-gemox regimen to the date of death or the last follow-up. PFS was measured from the date of the first administration of the P-gemox regimen to the date of disease progression or death from any cause. In the statistical design, patients were not censored at the time of transplantation.

The *χ*^2^ test was used for group comparisons of categorical variables. Survival analysis was performed using the Kaplan-Meier method, and curves were compared using the log-rank test. Two-tailed values of *P* < 0.05 were considered significant. Statistical analyses were performed by using SPSS 17.0 software.
